# Famotidine-Induced Rash and Eosinophilia: A Case Report

**DOI:** 10.7759/cureus.95133

**Published:** 2025-10-22

**Authors:** Abeera Junaid, Johann Graggaber

**Affiliations:** 1 General and Acute Internal Medicine, Cambridge University Hospitals NHS Foundation Trust, Cambridge, GBR; 2 Clinical Pharmacology, Acute Internal Medicine and Endocrinology, Cambridge University Hospitals NHS Foundation Trust, Cambridge, GBR

**Keywords:** allergy, cutaneous-drug eruption, drug-induced eosinophilia, famotidine, maculopapular rash

## Abstract

We report the case of a 77-year-old man who was admitted with a urinary tract infection and concurrent influenza A, who subsequently developed an unusual hypersensitivity reaction to famotidine. He initially received intravenous gentamicin followed by oral amoxicillin-clavulanic acid for the urinary tract infection. Oseltamivir and carbocisteine were prescribed for the flu. During admission, his proton pump inhibitor (PPI) was switched to famotidine due to hyponatremia. Four days later, he developed an itchy, erythematous maculopapular rash on his arms and legs. Initially localized to both extensor and flexural areas, the rash progressively spread to involve his torso over the next few days. A significant rise in eosinophils raised suspicion of a drug-induced reaction. Considering the timing of the rash and the patient’s lack of prior adverse reactions to penicillin, famotidine was identified as the most probable causative agent for the rash. The rash improved following discontinuation of famotidine, supporting the diagnosis of famotidine-induced hypersensitivity reaction.

## Introduction

Cutaneous eruptions are a frequent cause of hospital presentations and are also observed during inpatient admissions. Accurate diagnosis of drug-induced skin reactions can be challenging and requires thorough clinical history and physical examination.  Among the various causes of cutaneous eruptions requiring hospital care, common causes include adverse drug reactions, autoimmune conditions, and bacterial infections [1]. 

Famotidine, a histamine-2 receptor antagonist, is generally regarded as safe and well-tolerated. Nonetheless, rare cases of hypersensitivity reactions and anaphylaxis have been reported. Reporting drug-induced reactions is crucial for enhancing patient safety. It promotes vigilance among healthcare professionals when prescribing medications and helps in the early identification and management of adverse drug reactions.

According to the British Society for Allergy and Clinical Immunology (BSACI), 6.5% of hospital admissions are caused by adverse drug reactions and contribute to prolonged hospital stay in up to 15% of cases [2]. Immediate allergic responses such as anaphylaxis are immunoglobulin E (IgE) mediated, as they are caused by IgE against a specific allergen, whereas delayed reactions are usually non-IgE mediated, as they do not involve immunoglobulin E but may be mediated by other immune mechanisms, such as T-cell response; these may include delayed skin reactions like maculopapular rashes. Skin prick testing may support diagnosis in suspected IgE-mediated hypersensitivity; however, if the reaction is deemed to be non-IgE mediated, skin testing is not recommended. For delayed or T-cell-mediated reactions, patch testing or delayed intradermal testing is recommended [2].

## Case presentation

This 77-year-old man presented with symptoms of a urinary tract infection (UTI) related to a long-term suprapubic catheter. His past medical history included paraplegia from traumatic spinal cord injury, resulting in neurogenic bladder. His other comorbidities included prostate cancer, non-alcoholic steatohepatitis, and hypertension. Medications included simvastatin, omeprazole, amlodipine, chlorphenamine, pregabalin, duloxetine, baclofen, and goserelin.  

Based on his presentation, he was treated with IV gentamicin for 48 hours, followed by oral amoxicillin-clavulanic acid for seven days. On day two, the respiratory PCR done as part of the initial infection screen came back positive for influenza A; hence, oseltamivir was started for five days, and carbocisteine was prescribed for sputum clearance. Patient recovered well and was transferred to a medically fit-for-discharge ward where medical reviews are infrequent.  

**Figure 1:**
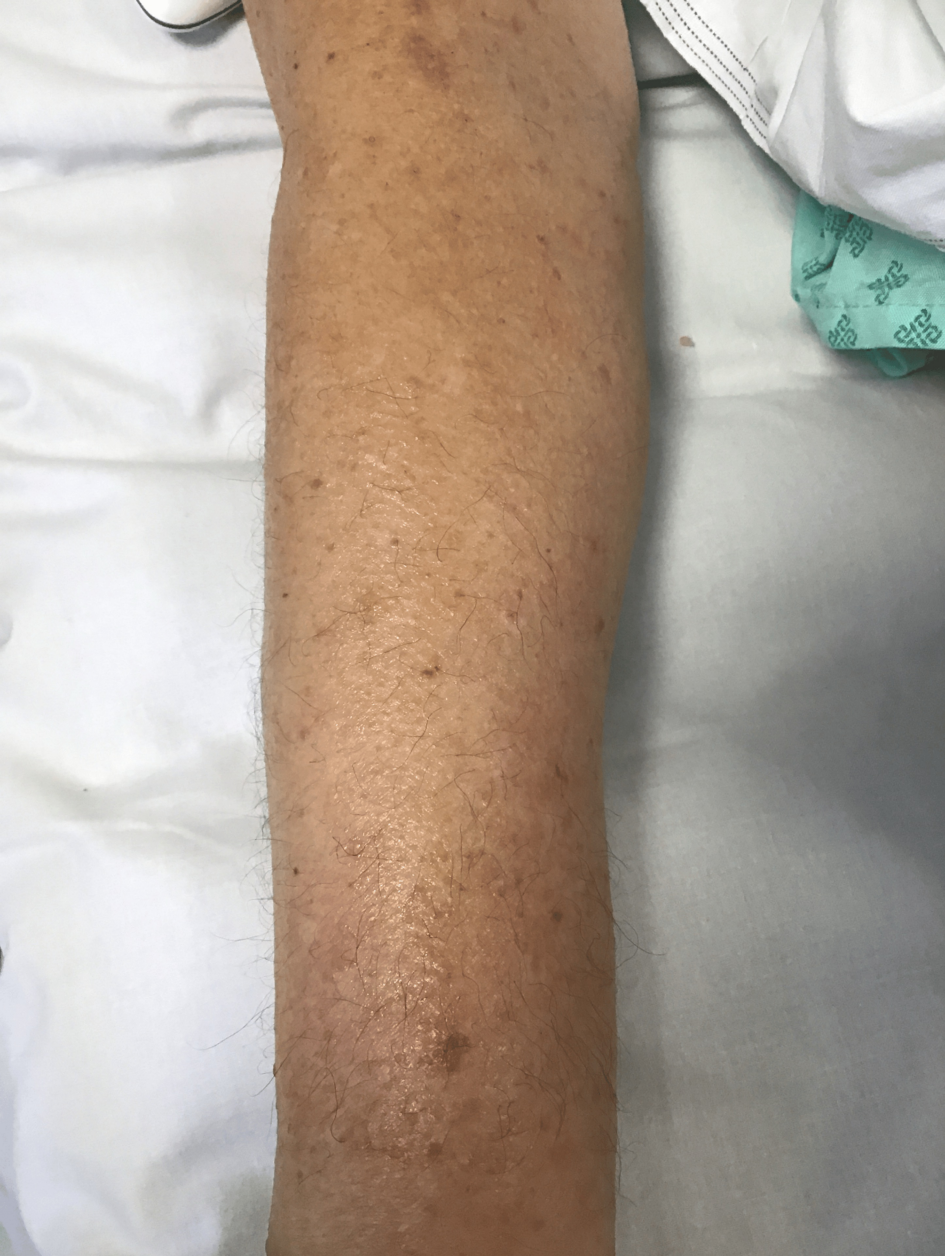
Erythematous maculopapular lesions on extensor surface of forearm

**Figure 2:**
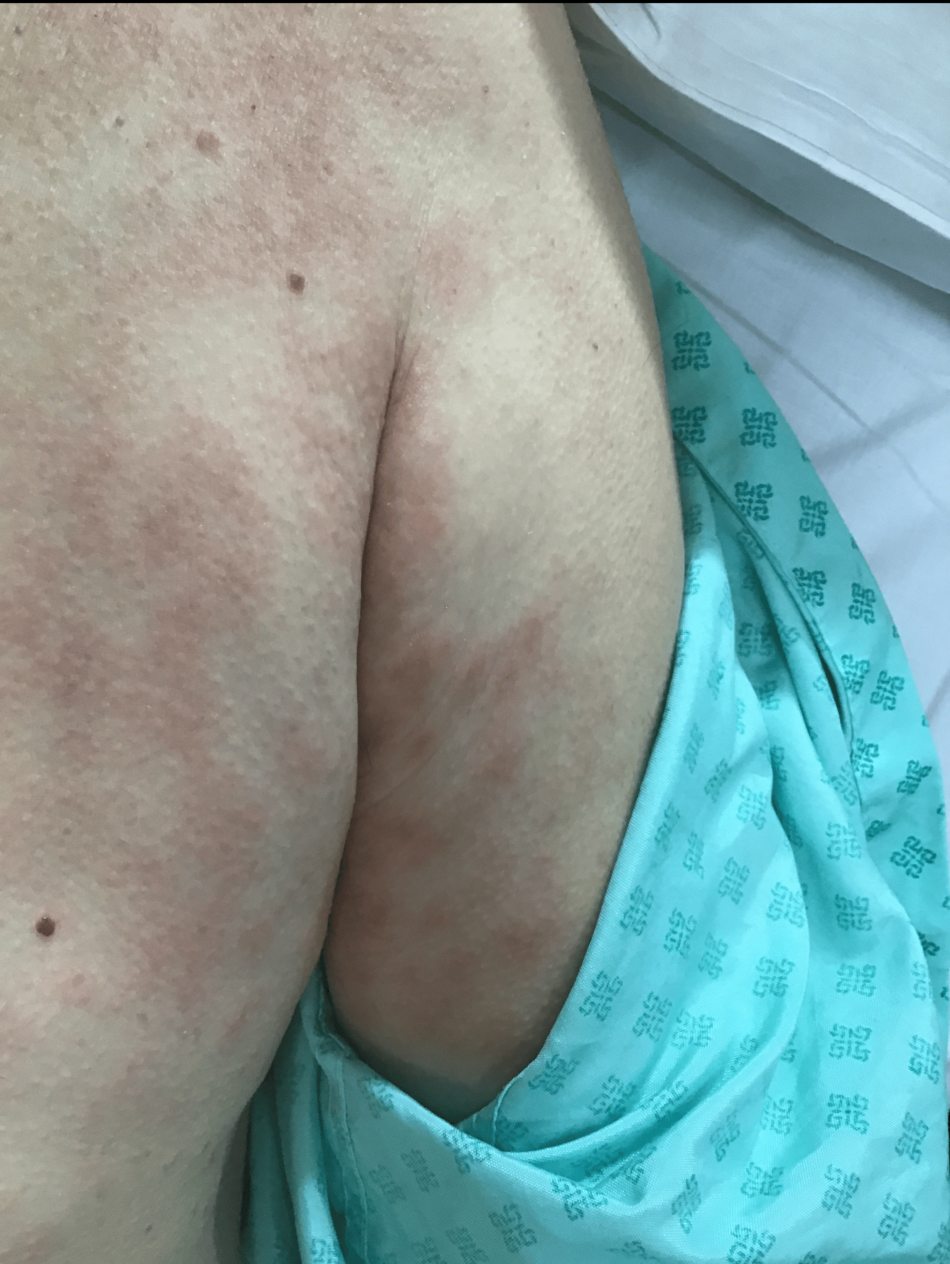
Erythematous maculopapular lesions involving upper arm and torso

**Figure 3:**
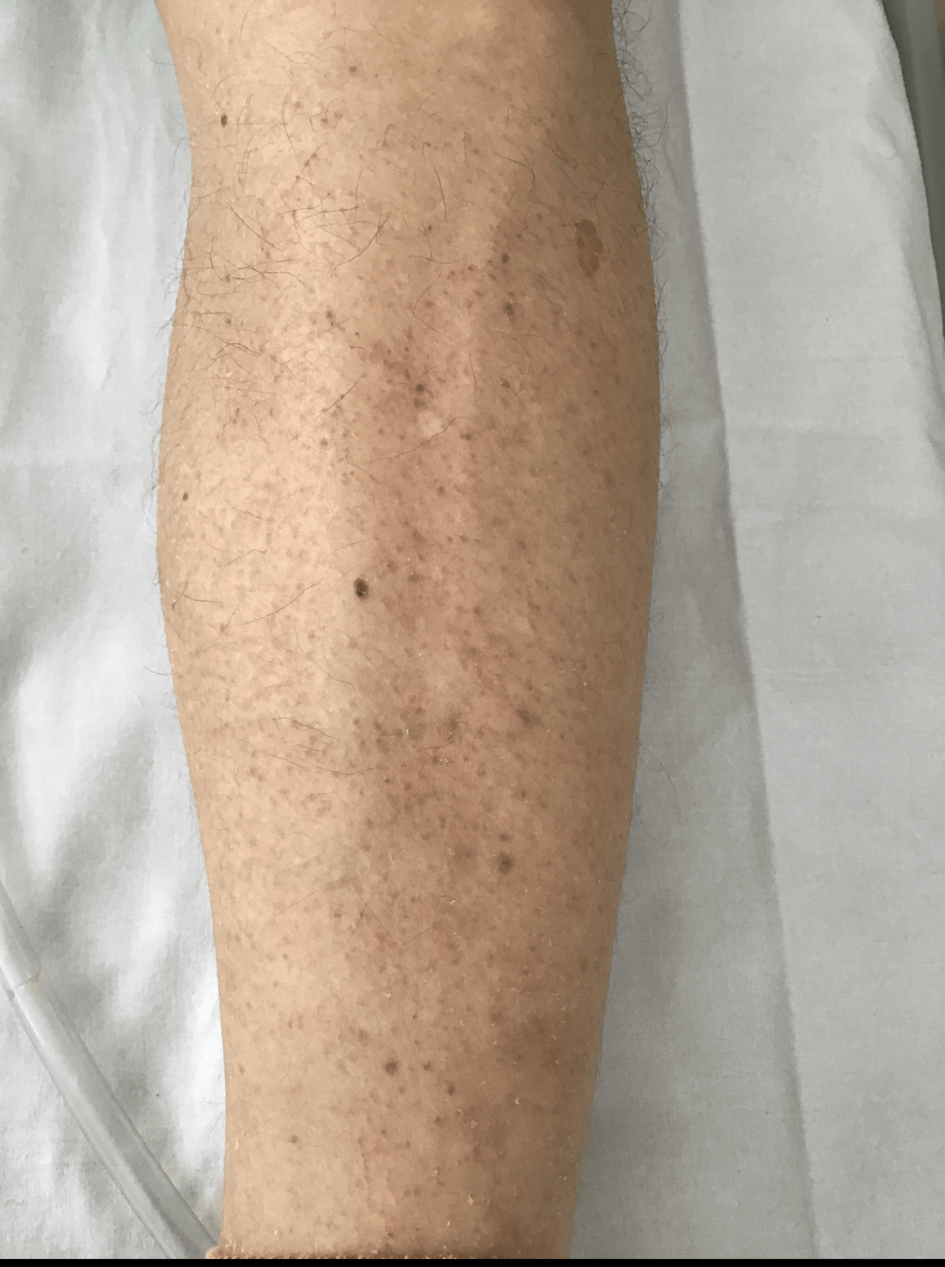
Multiple erythematous macules and papules on extensor surface of leg

During admission, he developed hypo-osmolar hyponatremia (Na⁺ 126 mmol/L; reference: 133-146 mmol/L). Based on further investigations and clinical probabilities, omeprazole was considered a potential cause and was replaced with famotidine. Four days after starting famotidine, the patient developed an erythematous, pruritic maculopapular rash with signs of excoriation involving the forearm (Figure 1), upper arm and torso (Figure 2), and lower leg (Figure 3). The rash was only reported to the medical team 10 days after the onset. Blood tests on the 10th day of rash onset showed eosinophilia at 1.69 × 10⁹/L (reference: 0-0.5 × 10⁹/L). Table 1 shows the trend of eosinophils corresponding to the day of starting famotidine.

**Table 1:** Eosinophil count corresponding to the day of starting famotidine

Number of days corresponding to start date of famotidine	Eosinophil count	Reference range
Day 4	0.60	0-0.5 × 10⁹/L
Day 7	0.66	0-0.5 × 10⁹/L
Day 9	0.69	0-0.5 × 10⁹/L
Day 15	1.69	0-0.5 × 10⁹/L
Day 16	2.81	0-0.5 × 10⁹/L
Day 17	3.37	0-0.5 × 10⁹/L
Day 18	3.28	0-0.5 × 10⁹/L
Day 19	2.86	0-0.5 × 10⁹/L
Day 25	0.89	0-0.5 × 10⁹/L
Day 45	0.52	0-0.5 × 10⁹/L

A dermatology consultation was sought, and an evaluation for allergic, inflammatory, autoimmune, and parasitic causes was carried out. Investigations included: repeat urine culture, stool microscopy for ova/parasites, serum protein electrophoresis, immunoglobulins, complement levels, and hepatitis screen. All of these investigations returned negative results (Table 2). 

**Table 2:** Summary of investigations ANA, antinuclear antibodies; dsDNA, double-stranded DNA; ELISA, enzyme-linked immunosorbent assay

Investigation	Result	Reference range
Antinuclear antibodies (ANA ELISA): dsDNA, ENA, and centromere	0.2	0.0-0.9 units
Rheumatoid factor	<10	0-13 IU/mL
Anti-neutrophil cytoplasmic antibody (ANCA)	Negative	Positive or negative
Complement C3	1.49	0.75-1.65 g/L
Complement C4	0.45	0.14-0.54 g/L
Serum protein electrophoresis	No monoclonal bands seen	-
Total protein levels	64	60-80g/L
Hepatitis B surface antigen	Negative	Positive or negative
Anti-Hbc core antibody	Negative	Positive or negative
Anti-Hbc total	Negative	Positive or negative
Blood film	Mild neutrophilic leukocytosis and eosinophilia	-
Fecal culture (for *Salmonella*, *Shigella*, *Campylobacter*, *Escherichia coli*, *Cryptosporidium*)	Negative	Positive or negative
Urine culture on admission	Negative	Positive or negative
Urine culture during admission	>10⁵ CFU/mL of *Pseudomonas aeruginosa*	Positive or negative

The patient had previously tolerated co-amoxiclav without adverse effects on multiple occasions. Moreover, penicillin-related rashes mostly cause immediate hypersensitivity reactions, which are IgE mediated [3]. Although repeated penicillin exposure may raise IgE-mediated allergy risk [4], no signs of an IgE reaction were observed in this case. Instead, a delayed cutaneous reaction suggested a non-IgE-mediated hypersensitivity. Furthermore, a review of the patient’s chart after discharge confirmed that he received penicillin after discharge without any adverse reactions.  

The eosinophilia could not be accounted for by the investigations mentioned above, making a drug-related cause a probable differential diagnosis. Moreover, bacterial infections are usually implicated in causing eosinopenia rather than eosinophilia. Eosinopenia has been deemed to be a useful marker to differentiate between infectious and non-infectious illnesses [5]. However, eosinopenia due to bacterial infection typically responds well to antibiotic treatment, often showing improvement within the first 24 hours, which was not the case in our patient [6].

## Discussion

Drug-induced cutaneous reactions may occur via immunological (allergic) or non-immunological mechanisms. The majority, however, are due to non-immunological pathways [7]. Identified risk factors for drug eruptions include viral infections, polypharmacy, previous hypersensitivity reactions, and positive family history of drug allergies [8].  

In this case, a pruritic, maculopapular rash developed shortly after the initiation of famotidine. As per the Primary Care Dermatology Society  (PCDS) of the United Kingdom, it usually takes four to 14 days for acute drug eruptions to appear after drug initiation [9]. In this case, the rash appeared four days after starting famotidine. Typical drug-induced reactions include morbilliform exanthema, urticaria, Stevens-Johnson syndrome (SJS), drug reaction with eosinophilia and systemic symptoms (DRESS), and erythroderma. The rash was associated with isolated peripheral eosinophilia and improved promptly after drug cessation, supporting a diagnosis of a non-IgE-mediated drug eruption. The absence of systemic features helped exclude more severe hypersensitivity syndromes such as DRESS or SJS.  

While famotidine is widely considered a safe and well-tolerated H₂-receptor antagonist, rare hypersensitivity reactions have been reported. A 2022 case series documented four COVID-19-positive patients who developed macular rashes on their limbs after taking famotidine; symptoms resolved upon discontinuation [10]. Anaphylactic reaction to famotidine was reported in 2010 in South Korea [11]. Furthermore, cross-reactivity between famotidine and other H₂-receptor antagonists, such as cimetidine and ranitidine, has also been established [11-12]. 

Oseltamivir was also considered, but a retrospective cohort study has not shown a consistent link between oseltamivir and generalized rash [13]. While isolated reports of oseltamivir-associated Stevens-Johnson syndrome and toxic epidermal necrolysis exist [14-16], our patient exhibited no mucosal involvement or systemic compromise, making this unlikely.  

Although the patient was concurrently receiving carbocisteine at the time the rash developed, he had previously received carbocisteine without any adverse reactions. Furthermore, an improvement in the patient’s eosinophilia was observed following the discontinuation of famotidine, despite the continuation of carbocisteine. Given the patient’s prior tolerance to carbocisteine and the temporal association between stopping famotidine and the clinical improvement, carbocisteine was not considered the likely causative agent.

While the presence of eosinophilia raised concern for DRESS syndrome, this diagnosis was excluded on clinical grounds. The patient was systemically well, apyrexial, and did not have lymphadenopathy or other organ involvement. 

The patient was treated with topical clobetasol propionate 0.05% ointment (initially once daily, tapered over six weeks), emollients, and oral fexofenadine 180 mg daily for pruritus. No systemic corticosteroids were required as the rash improved after famotidine withdrawal.

## Conclusions

The patient was discharged one week after discontinuing famotidine, by which time the rash had started to improve significantly. He was reviewed in the medical ambulatory care unit one week after discharge. By then, the rash had almost completely resolved, and he was systemically well. His eosinophil count had decreased to 0.89 × 10⁹/L (reference range: 0-0.5 × 10⁹/L). He was discharged with no further follow-up and was safety-netted to seek medical attention if he developed new symptoms.

This case adds to the limited literature on famotidine-induced cutaneous adverse reactions and highlights the importance of vigilance even with drugs not typically associated with hypersensitivity responses.
